# Supplementary material for: Can "sponge city" pilots enhance ecological livability: Evidence from China
Source: PLoS One. 2024 Jun 6;19(6):e0297251. doi: 10.1371/journal.pone.0297251 (PMC11156270; doi:10.1371/journal.pone.0297251)
Supplement: Attachment — Submitted filename: Response to Reviewers.docx [file pone.0297251.s003.docx]

Can "Sponge City" Pilots Enhance Ecological Livability: Evidence from China

Response to Reviewers

Thanks very much for the valuable suggestions of reviewers, we have made the following modifications and improvements to the manuscript according to the prompts:

**Reviewer #1:**

**Firstly, in the abstract section, further revision is recommended.**

Response：Thank you very much for your suggestions on the abstract. Based on the suggestions, we have carefully discussed and revised the grammar and logic of the sentences in the manuscript.

**Secondly, Figure 1. Kernel density estimation of ecological livability. The picture is not clear enough. Please replace it.**

Response：Thank you very much for your suggestions on the Figure 1 of the manuscript. According to the reviewer's prompts, we have carefully revised the Figure 1. Due to the close distance between the lines and the difficulty in widening their distance,so we mainly adjusted the color and style of the lines to make the figure clearer.

In addition, we have uploaded all the data and code used in the manuscript to “Supporting Information”.
